# A Genome-Wide Association Study of Field and Seedling Response to Individual Stem Rust Pathogen Races Reveals Combinations of Race-Specific Genes in North American Spring Wheat

**DOI:** 10.3389/fpls.2018.00052

**Published:** 2018-01-30

**Authors:** Erena A. Edae, Michael O. Pumphrey, Matthew N. Rouse

**Affiliations:** ^1^Cereal Disease Laboratory, United States Department of Agriculture - Agricultural Research Service (USDA ARS), St. Paul, MN, United States; ^2^Department of Plant Pathology, University of Minnesota, St. Paul, MN, United States; ^3^Department of Crop and Soil Sciences, Washington State University, Pullman, WA, United States

**Keywords:** genome-wide association, stem rust, *Triticum aestivum*, major genes, multi-locus model

## Abstract

Stem rust of wheat caused by the fungal pathogen *Puccinia graminis* f. sp. *tritici* historically caused major yield losses of wheat worldwide. To understand the genetic basis of stem rust resistance in contemporary North American spring wheat, genome-wide association analysis (GWAS) was conducted on an association mapping panel comprised of 250 elite lines. The lines were evaluated in separate nurseries each inoculated with a different *P. graminis* f. sp. *tritici* race for 3 years (2013, 2015, and 2016) at Rosemount, Minnesota allowing the evaluation of race-specificity separate from the effect of environment. The lines were also challenged with the same four races at the seedling stage in a greenhouse facility at the USDA-ARS Cereal Disease Laboratory. A total of 22,310 high-quality SNPs obtained from the Infinium 90,000 SNPs chip were used to perform association analysis. We observed often negative and sometimes weak correlations between responses to different races that highlighted the abundance of race-specific resistance and the inability to predict the response of the lines across races. Markers strongly associated with resistance to the four races at seedling and field environments were identified. At the seedling stage, the most significant marker-trait associations were detected in the regions of known major genes (*Sr6, Sr7a*, and *Sr9b*) except for race QFCSC where a strong association was detected on chromosome arm 1AL. We postulated the presence of *Sr2, Sr6, Sr7a, Sr8a, Sr9b, Sr11, Sr12, Sr24, Sr25, Sr31*, and *Sr57* (*Lr34*) in this germplasm based on phenotypic and marker data. We found over half of the panel possessed three or more *Sr* genes, and most commonly included various combinations of *Sr6, Sr7a, Sr8a, Sr9b, Sr11, Sr12*, and *Sr57*. Most of these genes confer resistance to specific *P. graminis* f. sp. *tritici* races accounting for the prevalent stem rust resistance in North American spring wheat.

## Introduction

Stem rust of wheat (*Triticum aestivum* L.), caused by *Puccinia graminis* f. sp. *tritici* (*Pgt*) Erikss. & Henning, is potentially a destructive disease of wheat at the global scale (Singh et al., 2011). In North America, the use of resistant varieties coupled with efforts to eradicate the alternate host of *Pgt*, barberry (*Berberis vulgaris* L.) led to successful control of wheat stem rust (Jin and Singh, 2006). Unlike in the decades 1900–1960, major losses due to wheat stem rust have not recently occurred in the United States (Roelfs et al., 1993; Kolmer et al., 2007). *Pgt* isolates have been classified as races depending on their reactions to a total of 20 differential wheat lines with single stem rust resistance genes (Jin et al., 2008). *Pgt* races have been denoted by a five-letter code, such as TTKSK, that represents the reaction of a given *Pgt* isolate to the 20 wheat stem rust resistance genes in the differential set. Although there was a large decline in the number of *Pgt* races since 1960, race TPMKC was the most prevalent race in the United States until the late 1980s. After this time, the frequency of race TPMKC was low because of the use of resistance cultivars of both hard red winter and spring wheats that carried the stem rust resistance (*Sr*) gene *Sr6* (Kolmer et al., 2007). The most common *Pgt* race in the US since 2003 has been race QFCSC that is more avirulent compared to race TPMKC (Jin, 2005; Kolmer et al., 2007). Races QTHJC and RCRSC have also been observed at various frequencies in the US *Pgt* population (McVey et al., 2002). McVey et al. (2002) also noticed the decline of race TPMKC from 1997 to 1998 on wheat and the increase of race RCRSC in 1998. Based on the reaction of the four races to the wheat stem rust resistance genes in the differential set, race TPMKC is virulent to *Sr8a, Sr9e*, and *SrTmp* but avirulent to *Sr9a* and *Sr9b*, whereas RCRSC is virulent to *Sr9a* and *Sr9b* but avirulent to *Sr8a, Sr9e*, and *SrTmp* (Supplemental Table 1). QTHJC is the only race virulent to *Sr6* out of the four races. TPMKC is also the only race virulent to both *Sr9e* and *SrTmp* among the four races. In a population structure analysis of North American *Pgt* races conducted by Stoxen (2012), a Neighbor-joining analysis of 62 North American *Pgt* isolates (the four races TPMKC, RCRSC, QTHJC, and QFCSC were included) with 20 SSR markers assigned the isolates into 9 well-supported clades. The four races were placed in different clades. The pairwise genetic differentiation values (Rst values) among the nine groups were also significant at *P* < 0.05 (Stoxen, 2012). These values ranged from 0.188 (QFCSC-QTHJC groups) to 0.607 (TPMKC-RCRSC). In summary, both phenotypic and genotypic analyses indicated that these races are distinct from each other.

Spring wheat breeders in the North Central United States and the Prairie Provinces of Canada have aggressively selected for stem rust resistance resulting in the deployment of wheat cultivars with resistance to the prevalent races of *Pgt*. Widely grown cultivars that were selected specifically for their resistance to stem rust include “Thatcher” released in 1935 (Thatcher Wheat, 1936), “Chris” released in 1966 by the Minnesota Agricultural Experiment Station (Kolmer et al., 2007), and “Selkirk” released in 1953 (Martens and Dyck, 1989).

These cultivars served as important sources of stem rust resistance for the deployment of stem rust resistant wheat cultivars worldwide through the Green Revolution (Singh et al., 2008). The genetics basis of resistance to stem rust in these cultivars was characterized in multiple studies. Thatcher was found to possess *Sr* genes *Sr5, Sr9g, Sr12*, and *Sr16* (Luig, 1983; Knott, 2000). Selkirk had *Sr6, Sr7b, Sr9d, Sr17*, and *Sr23* (Roelfs and McVey, 1979). Chris possessed genes including *Sr5, Sr7a, Sr8a, Sr9g*, and *Sr12* (Singh and McIntosh, 1987). The genetic basis of stem rust resistance in more recently developed wheat cultivars in North America was postulated to be conferred by combinations of race-specific *Sr* genes (Kolmer et al., 1991) including *Sr6, Sr7, Sr9*, and *Sr17*. Though conventional spring wheat cultivars in North America have continued to be selected for resistance to stem rust, the genetic basis of resistance in these cultivars has not been elucidated.

The emergence of a new *Pgt* race virulent to several deployed *Sr* genes, commonly known as Ug99, presented global concerns which has resulted in thorough phenotypic characterization of available genetic resources for resistance (Jin et al., 2007; Rouse et al., 2011; Rahmatov et al., 2016), characterization of races in the Ug99 race group including TTKSK (Pretorius et al., 2000; Jin et al., 2008, 2009; Newcomb et al., 2016), identification of stem rust resistance loci (Hiebert et al., 2010; Yu et al., 2011, 2012, 2014; Haile et al., 2012; Letta et al., 2013, 2014; Njau et al., 2013; Singh et al., 2013; Periyannan et al., 2014; Zurn et al., 2014; Babiker et al., 2015, 2016; Basnet et al., 2015; Chen et al., 2015; Dunckel et al., 2015; Kumssa et al., 2015; Nirmala et al., 2016), *Sr* gene cloning (Periyannan et al., 2013; Saintenac et al., 2013; Mago et al., 2015; Steuernagel et al., 2016), and the development of Ug99-resistant wheat cultivars (Njau et al., 2010; Singh et al., 2015).

A total of 250 contemporary spring wheat cultivars and advanced breeding lines from North America were evaluated for response to Ug99 stem rust in Africa and assessed with molecular markers to facilitate GWAS (Bajgain et al., 2015; Singh et al., 2015). The response of these 250 lines to North American *Pgt* races was not characterized and thus no information was available on correlation of resistance to Ug99 and resistance to North American *Pgt* races.

The goal of the current study was to determine the genetic basis of resistance in North American spring wheat to multiple races of *Pgt* in seedling and field tests. Through this goal, we wanted to (1) test if resistance to North American races of *Pgt* is correlated to resistance to Ug99, (2) identify markers linked to stem rust resistance genes effective to North American *Pgt* races, and to (3) postulate the presence of *Sr* genes effective to North American *Pgt* races in North American spring wheat.

## Materials and methods

### Study materials and phenotyping

The Triticeae Coordinated Agricultural Project (TCAP) spring wheat association mapping panel consisted of 250 wheat lines from breeding programs in the U.S (69.6%), Agricultural and Agri-Food Canada (20.4%), and the International Maize and Wheat Improvement Center (CIMMYT, 10%; Table 1). The panel was assessed for response to four *Pgt* races: QFCSC (isolate 95MN1080), RCRSC (isolate 00MN99C), QTHJC (isolate 0069MN399), and TPMKC (isolate 74MN1409) for 3 years (2013, 2015, and 2016) in the field and in a greenhouse for seedling responses. Each isolate was originally collected in Minnesota, and the reaction of the four Pgt races is given in Supplemental Table 1. The field evaluations were conducted at the Rosemount Research and Outreach Center of the University of Minnesota in Rosemount, MN. Each year, four separate field nurseries were maintained at Rosemount. In each nursery, spreaders were inoculated with a different *Pgt* isolate. The location of each nursery was no closer than 500 m to another nursery in order to maintain adequate isolation of *Pgt* races at Rosemount (Roelfs, 1972). Each year, the four *Pgt* isolates were randomly matched to specific nursery locations at Rosemount. The wheat lines were planted in 1 m long rows and replicated twice, in separate blocks, in each year, and infection response (IR) (Roelfs et al., 1992) and disease severity (SEV) on the modified Cobb scale (Peterson et al., 1948) were recorded twice between milky-ripe and soft-dough growth stages. The average of the two records within each year and race were used for subsequent data analyses. The 1 m long rows were planted perpendicular to single rows of a mixture of stem rust susceptible wheat varieties “Morocco” and “Baart” used as stem rust spreaders. The spreaders were planted 1–2 weeks prior to the experimental plots. Stem rust epidemics in each nursery were initiated by inoculating a light mineral oil suspension of *Pgt* urediniospores onto the spreader rows using an Ulva+ sprayer (Micron Sprayers Ltd., Bromyard, UK). A unique sprayer was used for each *Pgt* isolate. Two inoculations of each nursery were conducted in between booting and heading growth stages of the spreader rows.

**Table 1:** Sources of wheat lines in the TCAP spring wheat association mapping panel.

**Origin/source**	**No. of lines**
Ag-Canada Alberta	10
CIMMYT	25
Ag-Canada Manitoba	13
Montana State University (MSU)	25
Ag-Canada Saskatchewan	28
South Dakota State University (SDSU)	30
University of California-Davis (UCD)	34
University of Idaho (UI)	32
University of Minnesota (UMN)	27
Washington State University (WSU)	26

Field IR, SEV, and seedling infection types (IT) were subjected to analysis of variance. Since the year effect was not significant, best linear unbiased predictors (BLUPs) were calculated in SAS v. 9.3 software (SAS Institute, 2011), and combined data across years for IR and SEV were used for GWAS analysis. Categorical IR classes were converted to a linear 0–9 scale (Gao et al., 2016). The seedling stage IT evaluation for the same isolates of the four races used in the field was performed according to conditions described in Rouse et al. (2011) using the 0 to 4 Stakman IT scale (Stakman et al., 1962) and converted to a 0–9 linear scale (Zhang et al., 2014; Gao et al., 2016) for subsequent analyses. Two replications of each isolate were evaluated on the 250 lines. Pearson correlation coefficients among races were calculated both for seedling and field responses.

### SNP genotyping and data filtering

All of the 250 lines were previously genotyped with 90,000 gene-based SNPs using a custom Illumina Infinium iSelect bead chip assay (Illumina Inc., Hayward, CA; Wang et al., 2014). The genotypic data were downloaded from the Triticeae Toolbox (https://triticeaetoolbox.org/wheat). All SNPs with missing values above 20%, heterozygosity level > 2%, and minor allele frequency (MAF) < 5% were removed. Lines with 10% missing values across the SNPs were also removed (nine accessions were eliminated). The final filtered data set consisted of 22,310 SNPs for 241 lines. The remaining missing data points were imputed with the LD-KNNI imputation algorithm (Money et al., 2015) in TASSEL 5 (Bradbury et al., 2007).

### Linkage disequilibrium (LD), population structure, and kinship analyses

Intra-chromosomal LD between all possible pairwise comparisons of SNPs was calculated as squared allele frequency correlation (*r*^2^) in Synbreed R package (http://synbreed.r-forge.r-project.org/). The pattern of LD decay over genetic distance was previously reported for this population (Bajgain et al., 2015). We calculated the pairwise LD values between SNPs to check whether QTL/gene-associated markers were in LD and to select neutral markers for assessing population structure.

As the presence of population structure is one of the limiting factors in association mapping, we employed a Bayesian clustering approach using a model-based quantitative population structure assessment method implemented in STRUCTURE v.2.3.4 (Pritchard et al., 2000). A total of 1,575 SNPs that had LD values (*r*^2^) < 0.2 compared to all other SNPs were selected as neutral markers for population structure analysis. An admixture model with correlated allele frequency and a burn-in of 50,000 iterations followed by 100,000 Monte Carlo Markov Chain (MCMC) iterations was performed to test k values from k = 1 to 10. For each k, five independent runs were conducted to test the sampling variance of population structure inference. To determine the most likely number of clusters (k), the output from STRUCTURE was submitted to the web-based STRUCTURE HARVESTER (Earl and vonHoldt, 2012). The optimum number of clusters was determined based on the Δk statistics from the 2nd order rate of change in the log probability of the likelihood function between successive k values (Evanno et al., 2005). Genetic distance-based cluster analysis was also conducted using the “hclust” script for the Ward cluster method in R (R Core Team, 2016). A population kinship matrix based on the complete SNP data was calculated in GAPIT R package (Lipka et al., 2012).

### Association analysis

Marker-trait associations for the identification of chromosome regions associated with stem rust resistance were computed with the single-locus mixed linear model (SLMM) (Yu et al., 2006) that was implemented in GAPIT R package (Lipka et al., 2012) using a compressed mixed linear model (Li et al., 2014; Tang et al., 2016), and by a multi-locus mixed model (MLMM; Segura et al., 2012). For the two model selection criteria for MLMM, the extended Bayesian information criterion (EBIC) and multiple-Bonferroni criterion (mBonf), we used EBIC across the traits and races. The genome-wide *p*-values of the selected models were adjusted for false discovery rate (FDR) for the purpose of comparing results with that of the single-locus mixed model. In both models, population structure using the 1st 10 principal components (PCA) and genetic relatedness among individuals (K) were fitted as covariates in the respective mixed models. To declare a significant QTL in the single locus model, initially a more relaxed marker-wise *p*-value of ≤ 0.001 to identify candidate QTL was used. For multiple comparison adjustment, a false discovery rate (FDR) of 10%, was applied for both single and multi-locus models as a threshold for identifying robust QTL. Common QTL between races or traits were also considered as evidence of true QTL.

Finally, genes/QTL that each line possessed were postulated based on the phenotype and haplotype of markers associated with disease response. Several of the identified QTL were coincident with previously characterized stem rust resistance genes. Gene postulations were made for wheat stem rust resistance genes *Sr2, Sr6, Sr7a, Sr9b, Sr11, Sr12, Sr24, Sr25, Sr31*, and *Sr57*. Wheat lines were postulated to possess *Sr7a* based on (1) the presence of the resistance allele of *Sr7a*-associated (Bajgain et al., 2015; this study) marker *IAAV3545* (synonym *IWB34733*) and (2) the presence of a resistant IT in response to *Sr7a*-avirulent race TKTTF (Bajgain et al., 2015). Wheat lines were postulated to possess *Sr8a* based on (1) the presence of the resistance allele of *Sr8a*-associated (Bajgain et al., 2015) and linked (Hiebert et al., 2017) marker *kwh54* (synonym *Excalibur_c12085_276*; *IWB22036*), and (2) the presence of a resistant IT in response to *Sr8a*-avirulent race TRTTF (Bajgain et al., 2015). Wheat lines were postulated to possess *Sr11* based on (1) the presence of the resistance allele of *Sr11*-associated (Bajgain et al., 2015) and linked (Nirmala et al., 2016) markers *KASP_6BL_IWB73072* (synonym *Tdurum_contig67619_532*) and *KASP_6BL_IWB46893* (synonym *Kukri_c60966_205*) and (2) the presence of a resistant IT in response to *Sr11*-avirulent race TKTTF (Bajgain et al., 2015). Genes *Sr6* and *Sr9b* were postulated based on the results in the current study (see Results). For *Sr12*, we screened the population with the marker *NB-LRR3* from Hiebert et al. (2016). For the remaining genes (*Sr2, Sr24, Sr25, Sr31*, and *Sr57*), our gene postulations were based on previously described molecular marker data for the same population (Bajgain et al., 2015).

## Results

### Seedling stem rust response

The distribution of seedling IT scores based on the linearized 0–9 scale revealed that the majority of the tested lines were resistant (< 6, corresponding to less than IT “2+” on the 0–4 Stakman scale) to the four tested races (Supplemental Figure 1). However, the proportion of resistant entries was different among the races. For instance, only 1.2% of the lines were susceptible (>6) to race QFCSC and about 14% of the lines were susceptible to race QTHJC. The proportions for the remaining two races (TPMKC and RCRSC) fell within these two values. A total of 85% (206 out 241) of the lines showed resistance (IT < 6) to all four races whereas the proportion of resistant lines for combinations of three races varied from 85.5 to 91.3%. Similarly, the proportion of lines that showed resistance to two races ranged from 85.5 to 95.8%. Although the majority of the tested lines showed resistant ITs, analysis of variance (ANOVA) indicated that there were highly significant (*p* < 0.01) differences among the entries for all races (Supplemental Table 2). The phenotypic correlation coefficients among the four races were in the range of moderate (*r* = 0.47 between QTHJC and QFCSC) to high (*r* = 0.7 between TPMKC and RCRSC; Supplemental Figure 2).

### Field stem rust response

For field responses of the association panel, combined analysis of variance was conducted across 3 years (2013, 2015, and 2016) for IR and SEV since the effect of year was not significant (Table 2). Highly significant (*P* < 0.01) phenotypic variation was observed among the lines for all four races for both traits (Table 2). The frequency distribution of BLUP values for both IR and SEV were approximately normal with a slight shift toward resistance in some cases especially for IR (Supplemental Figures 3, 4). Since square root and logarithm transformation did not improve normality of the data, the original data was used for SEV and the linearized data was used for IR for subsequent analyses.

**Table 2:** Variance components for random variables for infection response (IR) and severity (SEV) across three environments (2013, 2015, and 2016).

**Subject**	**IR@**	**SEV@**
Genotype	0.01071^***^	69.95^***^
Genotype × Race	0.006516^***^	32.91^***^
Genotype × year	0.001370^**^	16.36^***^
Year	0.000045ns	25.23ns
Year × Race	0.001062ns	5.63ns
Bloc	0.000350ns	1.84^*^
Genotype(Year × Race)	0.002282ns	17.86^*^
Residual	0.006136ns	23.37^**^

@, Race significant at P < 0.05;

* significant at P < 0.05;

** significant at P < 0.01;

*** *significant at P < 0.0001*.

There was strong correlation between field IR and SEV recorded for each race. However, the correlation coefficients between races were either low or in most cases negative both for IR and SEV (Supplemental Figure 5). The correlation coefficients of seedling IT with field IR and SEV were moderate (0.37–0.45) for all races except for TPMKC where seedling IT was weakly correlated with field SEV. No significant correlations were identified between our data on these North American *Pgt* races compared to the field data of the response of this panel to Ug99 in Ethiopia and Kenya (Bajgain et al., 2015; Supplemental Figure 6).

### Population structure analysis

**Figure 1:**
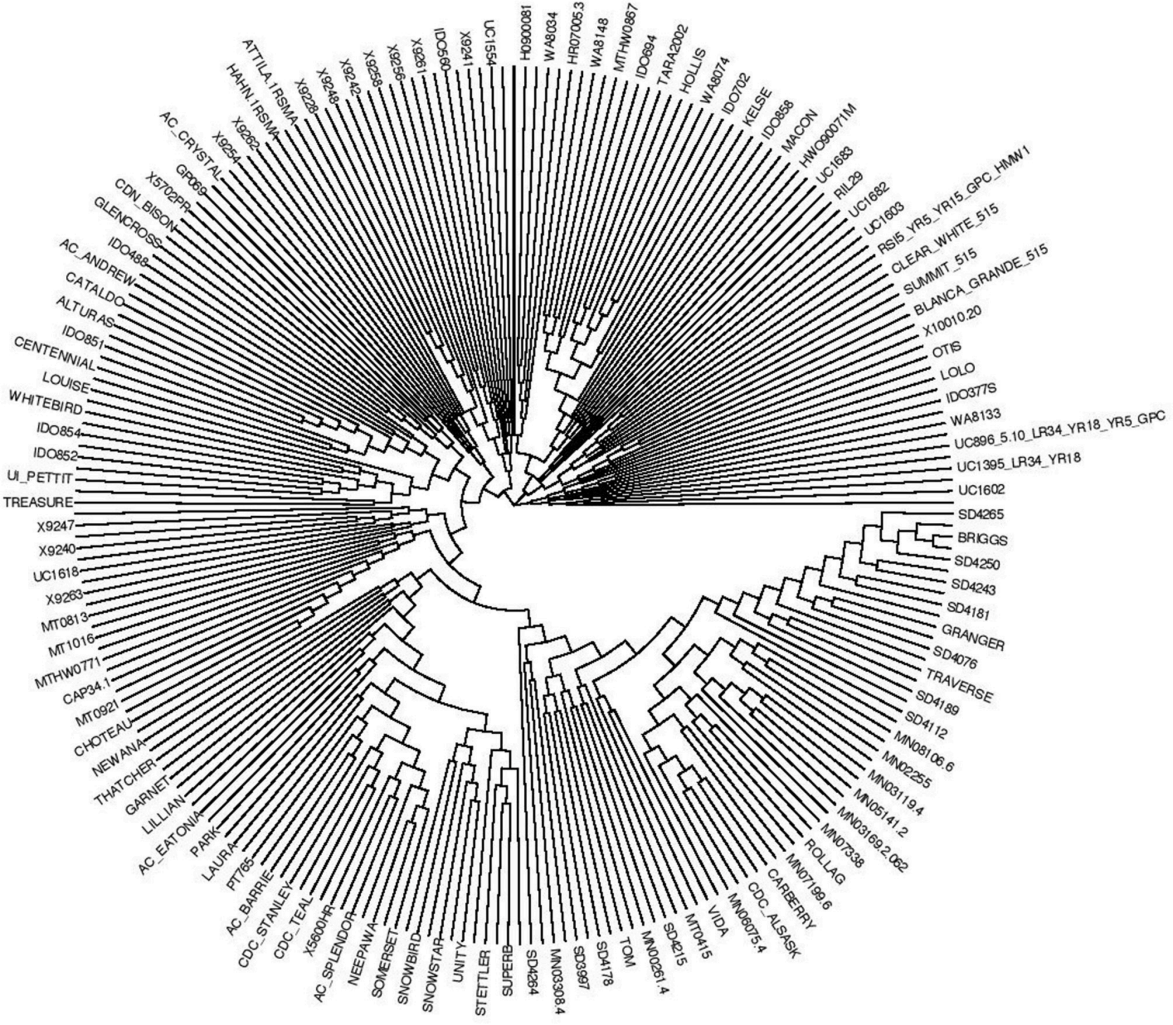
Genetic relationship among TCAP spring wheat association mapping germplasm.

The rate of change in the log probability (Δk) from the model-based population structure analysis indicated that the most likely number of subpopulations was two (Supplemental Figure 7). Among the five replicated runs at k = 2, the run with the highest posterior probability of the data (run seven for this analysis) was used as representative of the replications for downstream analysis. Although each line shared some amount of similarity to lines in both sub-populations, the two groups consisted of a similar number of lines with 106 (44%) lines in the 1st cluster (C_1_) and 135 (56%) lines in the 2nd cluster (C_2_) (Supplemental Figure 8). Majority of the lines in the C_1_ group were obtained from the North Central United States and Canada. Similarly, most of the lines in the C_2_ group were from the Western United States and CIMMYT. Few accessions from Canada (three from Alberta and two from Manitoba) and also eight lines from MSU were classified into the Western cluster (C_2_) (Supplemental Table 3). In distance-based cluster analysis using the whole marker data set (22,310 SNPs), the majority of the lines from the North Central United States and Canada grouped in the nearby cluster branches whereas lines from the Western United States and CIMMYT were also in another group of closest branches (Figure 1), largely supporting the results from the Bayesian-based clustering method.

### Association analyses for stem rust resistance

Association analysis was conducted for seedling infection type (IT), field infection response (IR), and field disease severity (SEV) separately for each race. The single-locus model output was validated by MLMM. The QTL detected with the single-locus model and confirmed by MLMM for four races at the seedling stage are presented in Table 3.

**Table 3:** Marker-trait associations that were identified for four races of *Puccinia graminis* f. sp. *tritici* in seedling assays.

**Race**	**Marker**	**Chromosome**	**Position (cM)**	***R*^2^ (%)**	***p*-value**	**Gene postulated**
QFCSC	*Excalibur_c1066_303*	1A	102.32	9.14	3.32 × 10^−5^	New QTL
QTHJC	*IWB17135*	2D	40.05	9.61	7.17 × 10^−7^	*Sr6*
TPMKC	*IWA2415*	2D	40.05	7.89	1.102 × 10^−5^	*Sr6*
	*IWB1190*	2B	119.07	9.05	3.47 × 10^−6^	Unknown
RCRSC	*IWA1505*	4A	145.19	12.34	1.84 × 10^−7^	*Sr7a*

### Association analysis for seedling response

**Figure 2:**
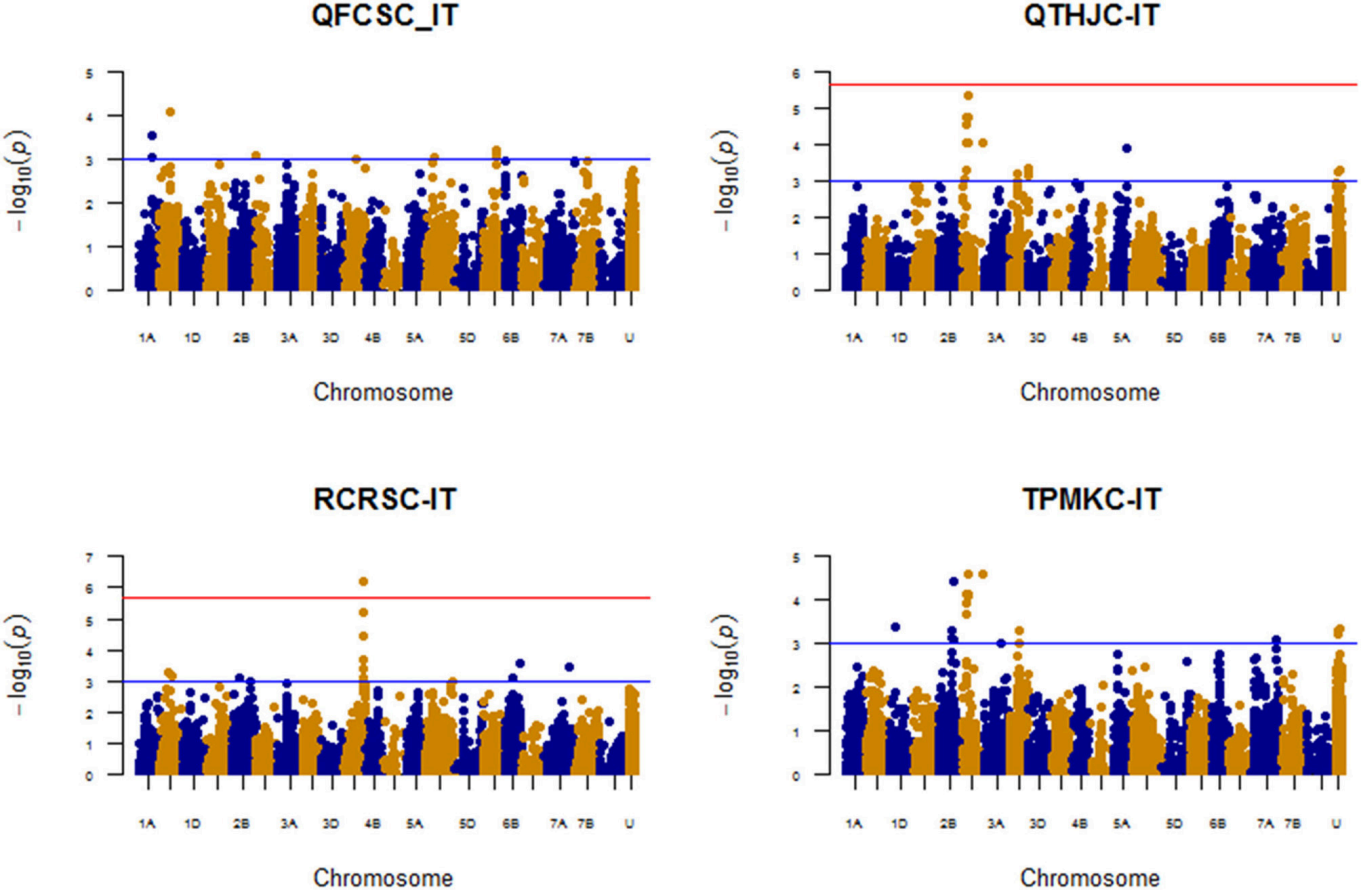
Manhattan plots for response to four races based on seedling infection type genome-wide association analysis.

**Figure 3:**
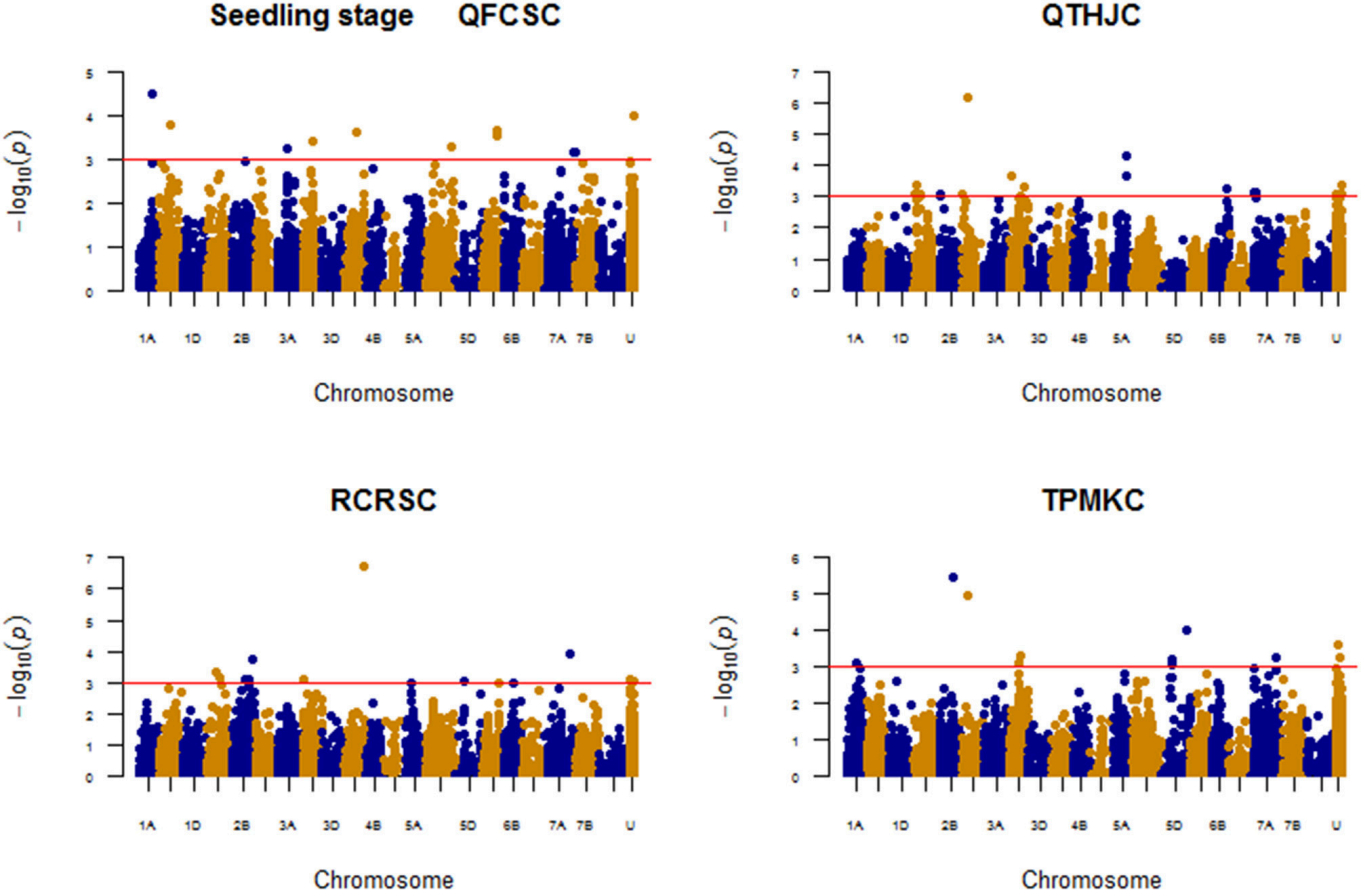
Manhattan plots for response to four races based on seedling infection type with MLMM model.

The reactions of the TCAP association mapping panel to the four *Pgt* races (QFCSC, QTHJC, RCRSC, and TPMKC) were used to identify chromosome regions associated with resistance. The compressed single-locus model that included kinship and the 1st 10 principal components accounted for the population structure well (Supplemental Figure 9). The number of marker-trait associations (MTAs) ranged from 11 for QFCSC to 28 for QTHJC (Supplemental Table 4, Figure 2) with a total of 79 candidate MTAs at *P* < 0.001 for all four races at the seedling stage (69 unique SNPs on 14 chromosomes). The highest number of MTAs were located on chromosome 2D (38%) followed by chromosomes 3B (11.4%) and 4A (8.9%). A total of seven (8.9%) MTAs were from markers of unknown chromosome location. By categorizing MTAs within 5 cM as one QTL, we identified a total of 25 QTL regions for markers with known chromosome locations. After applying the more stringent threshold (FDR of 10%), a QTL detected for race QTHJC on chromosome arm 2DS (36.54–40.05 cM), and another QTL detected on chromosome arm 4AL for race RCRSC (144.38–145.19 cM) showed significant association signals. The MLMM model was applied to identify the most significant markers in the QTL regions for these two races (Figure 3). Marker *D_contig73920_552* (*IWB17135*) on 2DS (at 40.05 cM) was significantly associated with resistance against race QTHJC (FDR *P* < 0.1). This marker is in LD (average *r*^2^ = 0.74) with markers in the range of 34.15–40.05 cM. Similarly, marker *IWA1505* (4AL at 145.19 cM) showed strong association (FDR *P* < 0.1) with race RCRSC and explained 12.31% of the phenotypic variation.

None of QTL regions for races QFCSC and TPMKC passed the stringent multiple comparison threshold. However, the most significant QTL detected for QFCSC was on chromosome 1B [at position 90.60 cM with marker *Ku_c9909_1766* (*IWB40197*)], and for race TPMKC was detected on 2DS [at position 40.05 cM with markers *RAC875_c57_1178* (*IWB59246*) and *IWA2415*, and at position 152.84 cM with marker *IWA2414*]. With MLMM model, a QTL conferring resistance against QFCSC was detected on 1A (102.32 cM) with marker *Excalibur_c1066_303* (*IWB21700*) (*p-value* = *3.32E-05*) instead of 1B (90.60 cM). To resolve these conflicting results, we re-ran the analysis using another multi-locus model that was implemented in FarmCPU R package (Liu et al., 2016) and found that SNP marker on chromosome 1A (position 102.32 cM) was actually the most significantly (*p-value* = *3.63E-05*) associated marker with QFCSC resistance at the seedling stage. For race TPMKC, a model with two markers *Bobwhite_c18540_97* (*IWB1190*; 2B; 119.07 cM; *p-value* = 3.47E-06) and *IWA2415* (2D, 40.05 cM; *p-value* = 1.10E-05) was selected as optimum in the MLMM model. However, only the former was significant at FDR *p*-value of 10%. We used marker *Bobwhite_c18540_97* (*IWB1190*) in addition to haplotypes of three other markers associated with seedling resistance to race TPMKC on chromosome arm 2BL to postulate the presence of *Sr9b* that is likely the wheat stem rust resistance gene conferring the observed QTL (see section Discussion). We used marker *IWA2415* in addition to three other markers associated with seedling resistance to race QTHJC on chromosome 2D to postulate the presence of *Sr6*. Both markers jointly explained 16.94% of the phenotypic variation for race TPMKC.

**Figure 4:**
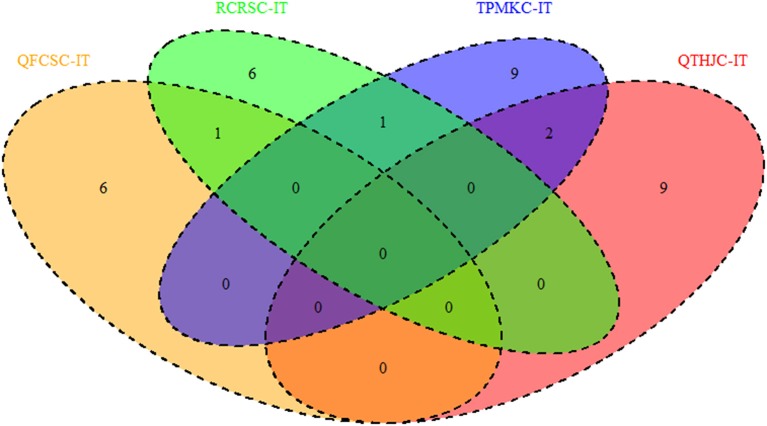
QTL regions shared among response to four races based on seedling infection type genome-wide association analysis.

In addition, MTAs detected across two or more races were also used as criteria for identifying reliable MTAs. Although there were no QTL shared among the four races, a total of four QTL regions were common between two races at the seedling stage. As shown in Figure 4, races QFCSC and RCRSC shared a common QTL region on chromosome 1B (90.60–94.40 cM); races QTHJC and TPMKC had two QTL in common on chromosome 2D (34.12–40.05 cM; 152.84 cM with marker *IWA2414*). Similarly, races RCRSC and TPMKC shared QTL on chromosome arm 7AL (map position 201.46–201.78 cM).

### Association analysis for field response

The field association analysis was conducted using combined data across 3 years (2013, 2015, and 2016) for infection response (IR) and disease severity (SEV) for each of the four *Pgt* races (RCRSC, QTHJC, TPMKC, and QFCSC) using both single-locus mixed model and MLMM.

### Race: QFCSC

**Figure 5:**
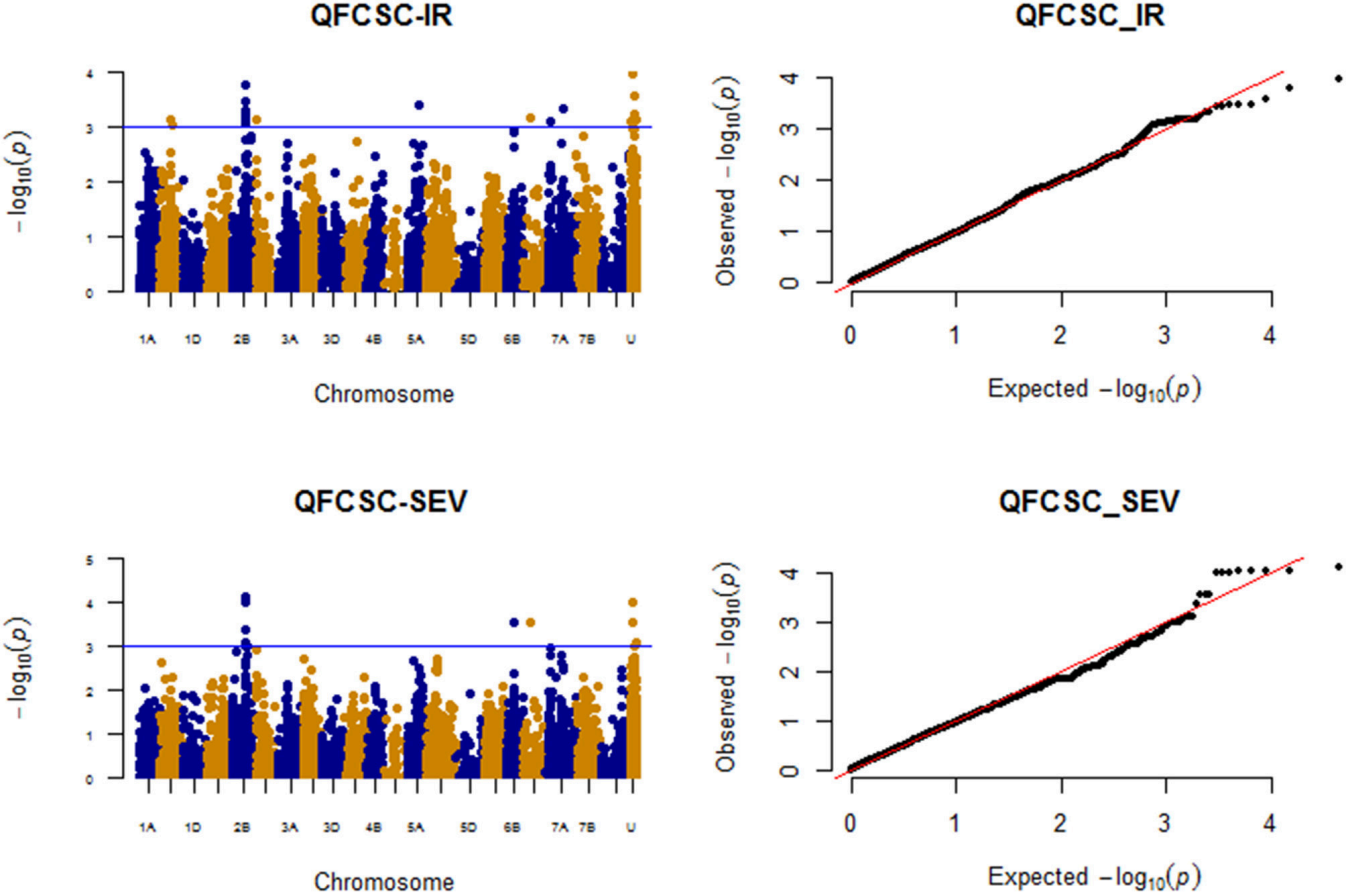
Manhattan plots for response to race QFCSC field data (IR and SEV).

The markers associated with resistance against race QFCSC are displayed on Figure 5.

#### Infection response (IR)

A total of eight significant (*P* < 0.001) QTL regions were identified on six chromosomes (1B, 2B, 2D, 5A, 6D, and 7A) after MTAs within 5 cM were merged together. Another eight unmapped markers also showed significant associations (Supplemental Table 5). None of the QTL regions were significant at FDR of 10%, but unmapped marker *IAAV2323* showed relatively strong association (*p*-value < 0.0001) followed by markers *GENE-0966_173* (*IWB32165*) and RAC875_c15396_90 on chromosome arm 2BL. Applying MLMM model, these three markers showed strong associations (FDR *p* < 0.1) for race QFCSC IR.

#### Disease severity (SEV)

Four chromosome regions on chromosomes 2B (108.45–110.87 cM; 119.07 cM), 6B (61.78 cM), and 6D (63.58 cM) were significantly associated with QFCS-SEV at *P* < 0.001; four unmapped markers were also significantly associated at this threshold level. With respect to QTL regions/MTAs common across IR and SEV for QFCSC, the QTL on chromosomes 2B (108.45–110.87 cM), 6D (63.58 cM with marker *CAP11_c4727_205*), and unmapped SNPs *Ex_c47157_508*, Tdurum_contig12459_405and *IAAV2323* were significantly associated with both IR and SEV for QFCSC (Figure 5; Supplemental Table 5). With MLMM, a model with markers *Excalibur_c76665_98* (*IWB28807*; 2B; 109.25 cM) and *IAAV2323* was found optimum, and both markers were significant at FDR *p*-value of 10% similar to associations detected with IR for QFCSC. These two markers jointly explained 15.94% of the phenotypic variation. The unmapped marker *IAAV2323* is in linkage equilibrium (*r*^2^ < 0.1) with other mapped markers detected in marker-trait associations in this study.

### Race: QTHJC

#### Infection response (IR)

**Figure 6:**
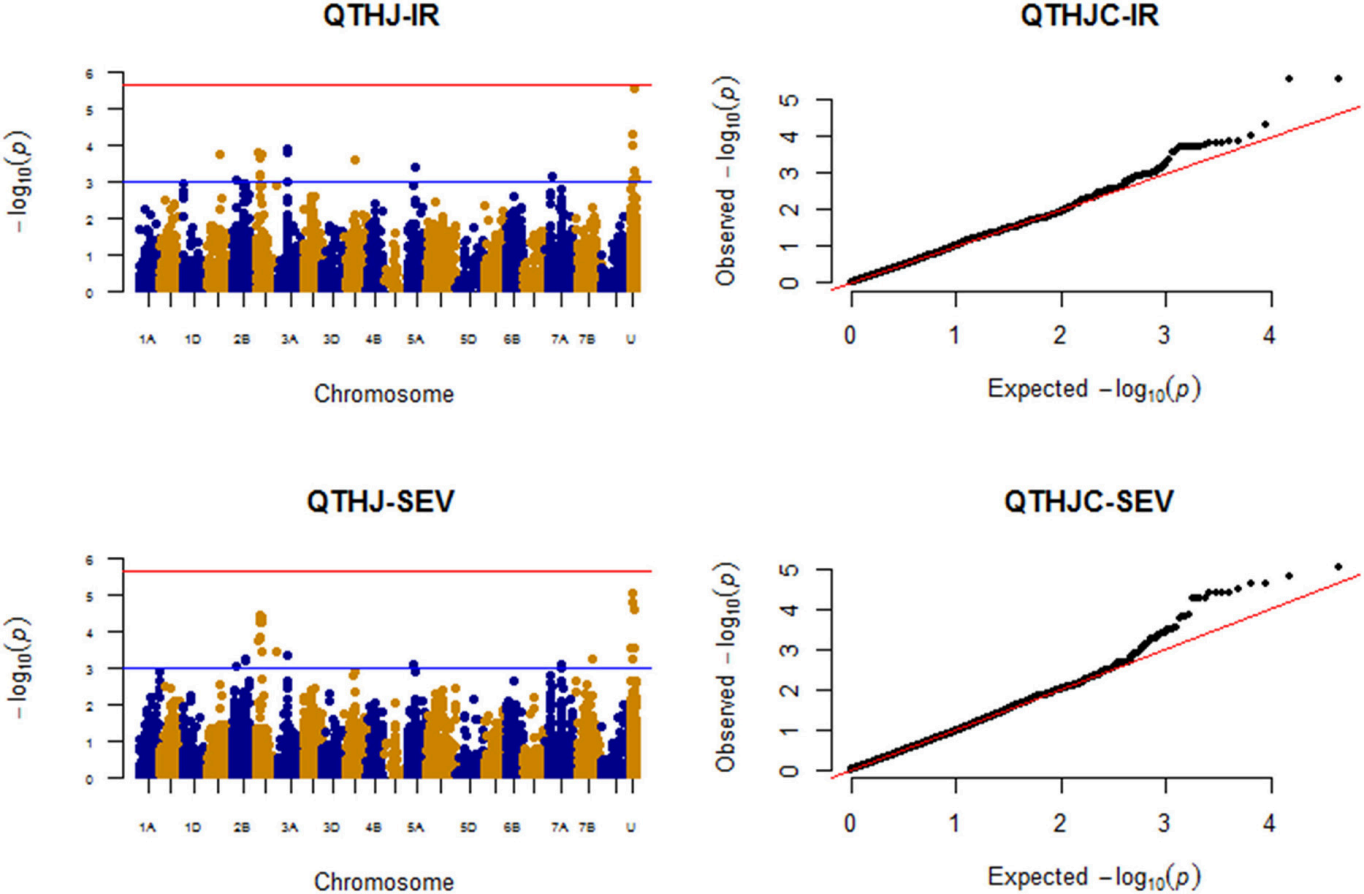
Manhattan plots for response to race QTHJC field data (IR and SEV).

As shown on Figure 6, a total of 15 QTL were identified and eight of them corresponded to markers with known chromosome positions on chromosomes 2A (101.97 cM), 2B (48.54 cM), 2D (22.46 cM; 34.15–40.05 cM), 3A (85.39–86.66 cM), 4A (74.51 cM), 5A (62.72 cM), and 7A (65.41 cM). Two unmapped markers *IWA760* and *RAC875_c7319_195* were significant at the FDR adjusted *p*-value of 10%, and each of these two markers explained about 8% of the phenotypic variation. In the MLMM, the selected optimum model consisted of two unmapped markers *IWA760* (*P* = 5.64E-07) and *Excalibur_c3574_607* (*P* = 2.07E-05) that were strongly associated with resistance against QTHJC based on IR with the former SNP marker passed multiple test FDR *P* < 0.1. Both markers together explained 11.46% of the phenotypic variation. Marker *IWA760* was also identified as the most significantly associated with QTHJC resistance with single-locus mixed model.

#### Disease severity (SEV)

A total of 15 significant QTL were detected for QTHJC SEV and eight of these were distributed on six chromosomes: 2B, 2D, 3A, 5A, 7A, and 7B. Four of the seven unmapped markers Excalibur*_rep_c69347_697, Excalibur_c3574_607, IWA760*, and *RAC875_c7319_195* showed significant association at FDR *p*-value of 10%. Similarly, a QTL region on chromosome arm 2DS (34.15–40.05 cM with nine MTAs) was also significant at FDR (*P* < 0.1). The QTL on chromosomes 2B (48.54 cM), 2D (22.46 cM), 2D (34.15–40.05 cM), and 3A (85.39–86.66 c) were common QTL between IR and SEV for QTHJC. Unmapped markers such as *Excalibur_c3574_607, Excalibur_rep_c69347_697, IWA760, RAC875_c7319_195*, and *RAC875_rep_c72596_466* were also shared between IR and SEV (Figure 6). From MLMM, three unmapped markers *IWA760* (*p-value* = *4.50E-07*), *Excalibur_c3574_607* (*p-value* = *1.45E-07*), and *RAC875_rep_c72596_466* (*p-value* = *4.68E-06*) were strongly associated with QTHJC based on SEV data with all three together explaining 22.33% of the phenotypic variation. All three of them showed significant associations at FDR *p-value* of 10%. The LD among these three markers is very negligible (*r*^2^ < 0.01) indicating that they are in linkage equilibrium and likely represent different QTL.

### Race RCRSC

**Figure 7:**
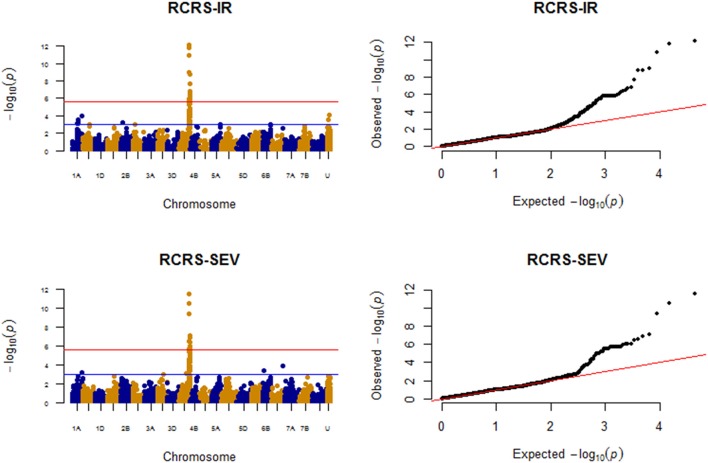
Manhattan plots for response to race RCRSC field data (IR and SEV).

MTAs detected for race RCRSC are shown in Figure 7.

#### Infection response (IR)

Twelve QTL were significantly associated with IR to RCRSC. Out of these, QTL on chromosomes 1A (at position 94.46 cM; 142.31 cM), 4A (142.29–147.53 cM; 149.53–153.00 cM; 163.83–164.13 cM) and two unmapped SNPs, *IWA2771* and *Kukri_c10641_584* were significant at FDR *p-value* < 0.1 (Figure 7; Supplemental Table 6). A maximum phenotypic variation of 17.45% was explained by SNP *IAAV3545* (4A; 144.37 cM). A model with marker *IAAV3545* (4A, 144.38 cM; *P* = 3.17E-20) and *Excalibur_c15048_488* (*IWB22544*) on chromosome 2D (37.62 cM; *P* = 2.97E-06) was selected as the optimum model in MLMM. The two SNPs together explained 26.40% of the phenotypic variation. Marker *IAAV3545* showed an exceptionally low *p-value* that was significant at the Bonferroni threshold level (*P* = 2.24E-06) and explained 21.53% of the phenotypic variation by itself.

#### Disease severity (SEV)

A total of seven QTL regions were significant (*P* < 0.001) for RCRSC-SEV. However, only QTL regions on 4A (142.29–147.54 cM; 151.33–152.99 cM; 163.83–164.13 cM) and 7A (6.38 cM) were significant at FDR *p-value* < 0.1. Similar to IR, the SNP *IAAV3545* (on 4A at position 144.37 cM) explained the most phenotypic variation (16.23%). QTL regions on chromosomes 1A with marker *Kukri_c44201_497* (*IWB45411*) (142.31 cM) and 4A (142.29–147.54 cM; 149.53–153.00 cM; 163.83–164.13 cM) were shared between RCRSC IR and SEV (Figure 7). With MLMM, a model with marker *IAAV3545* (4A, 144.38 cM; *P* = 2.95E-16) was found optimum, and this marker showed strong association (significant with Bonferroni *P* = 2.24E-06) with resistance against race RCRSC explaining 20.14% of the phenotypic variation. It was also mapped in the QTL region detected for RCRSC resistance with the single-locus mixed model.

### Race TPMKC

**Figure 8:**
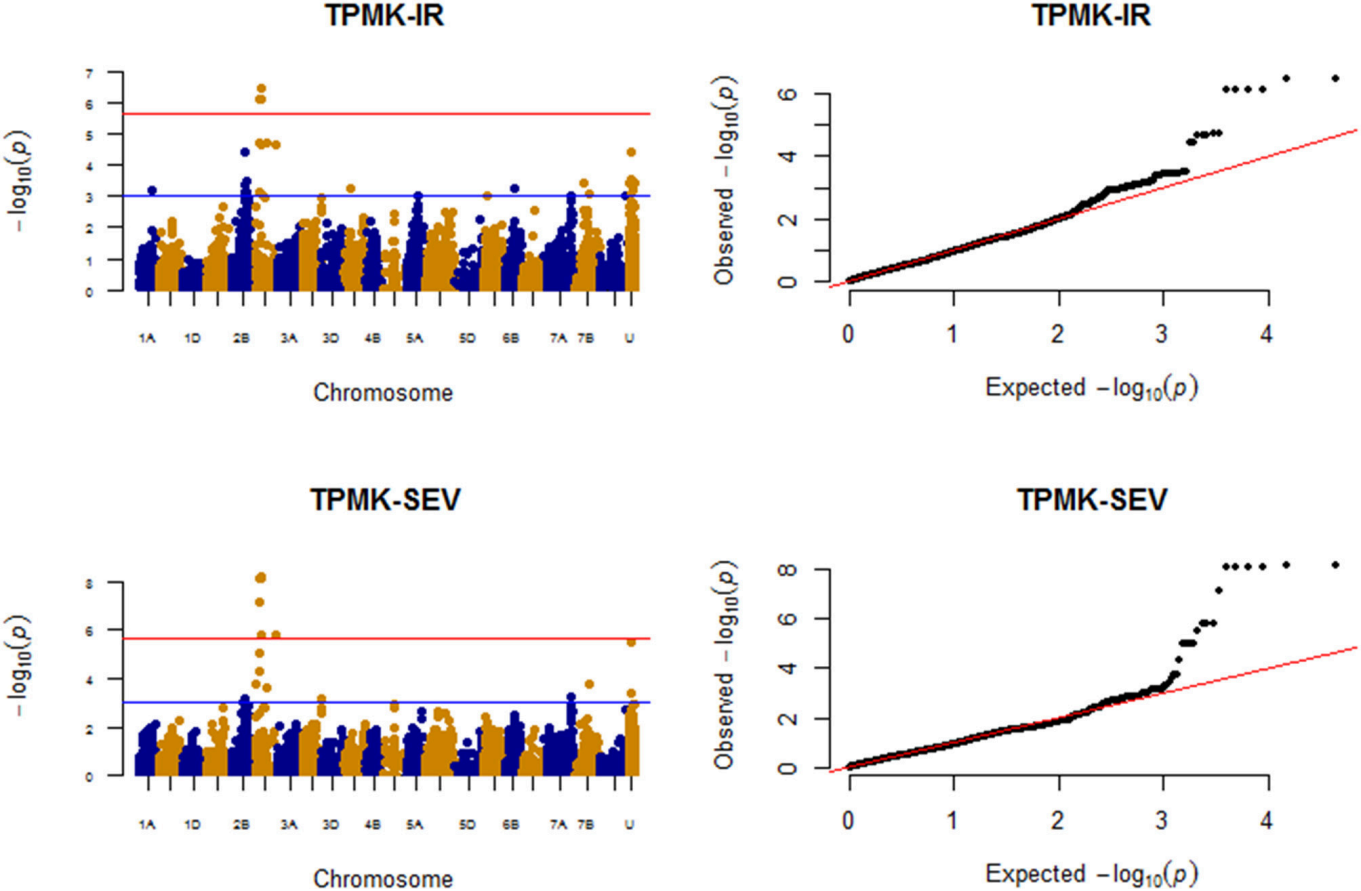
Manhattan plots for response to race TPMKC field data (IR and SEV).

The MTAs detected for resistance against race TPMKC based on both IR and SEV are indicated in Figure 8.

#### Infection response (IR)

A total of 13 QTL regions (excluding unmapped marker MTAs) were significantly associated with TPMKC IR at *P* < 0.001. However, only QTL on chromosomes 2D (80.41 cM), 2D (36.54–40.05 cM), 2B (115.01 cM) and unmapped SNP *Excalibur_c60964_203* were significantly associated with resistance against TPMKC (Figure 8; Supplemental Table 7) based on FDR of 10%. Applying MLMM, a model with four markers was found optimum for resistance against race TPMKC. Out of these markers, *D_contig73920_552* (*IWB17135*) on 2D (40.05 cM; *P* = 1.41E-09) and *Excalibur_c60964_203* (unmapped, *P* = 5.20-07) were significant with the Bonferroni test. Both markers jointly explained 18.46% of the phenotypic variation.

#### Disease severity (SEV)

Chromosomes 2B, 2D, 3B, 7A, and 7B harbored QTL regions that were significantly associated with resistance against TPMKC based on disease severity. Two QTL regions on chromosome 2D (34.15–40.05 cM; 152.84 cM) and unmapped SNP *IWA2414* passed the multiple comparison test (FDR *p-value* < 0.1). The former was strongly associated with resistance against TPMKC both with IR and SEV. However, with relaxed threshold level (*P* < 0.001), there were a total of 10 QTL regions that were shared by IR and SEV for race TPMKC (Figure 8). With MLMM analysis, a model with two SNPs, *D_contig73920_552* (*IWB17135*; 2D, 40.05 cM) and *IWA8141* (2B, 109.53 cM) were found optimum for SEV. Marker *D_contig73920_552* (*IWB17135*; *P* = 1.12E-10) was significantly associated at Bonferroni threshold level *P* < 2.24E-06 explaining 13.28% of the phenotypic variation.

**Figure 9:**
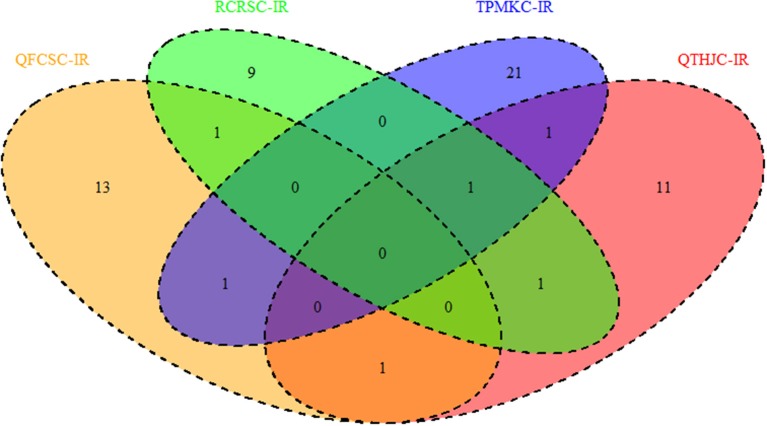
QTL regions shared among response to four races based on field IR.

**Figure 10:**
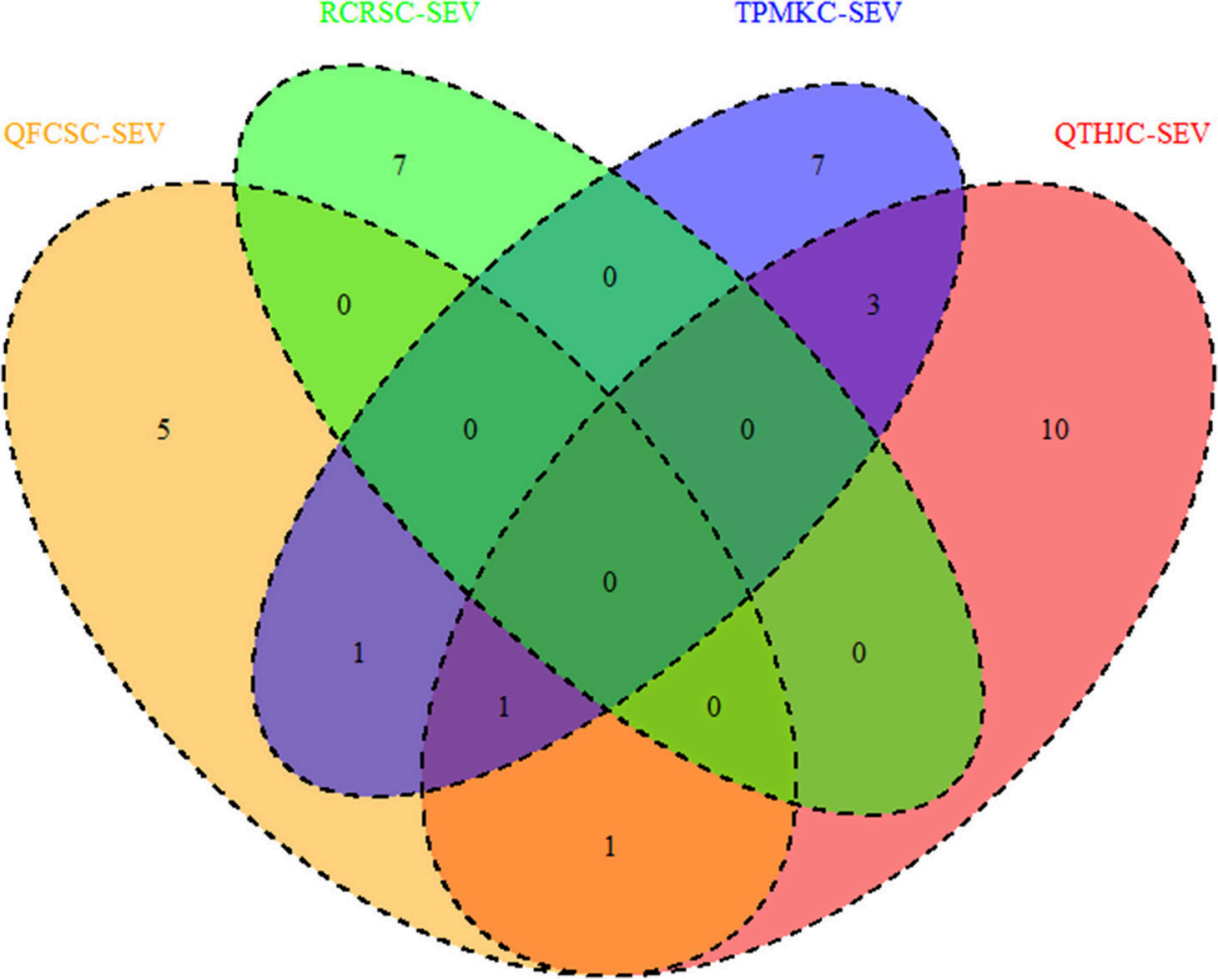
QTL regions shared among response to four races based on field SEV.

Generally, none of the QTL regions were common across the four races with IR, but there was a QTL region (34.15–40.45 cM) on chromosome arm 2DS that was shared among races QTHJC, RCRSC, and TPMKC (Figure 9). Similarly, one QTL region on chromosome 2B (108.45–110.87 cM) was common among QTHJC, QFCSC, and TPMKC (Figure 10).

### Gene postulation

**Figure 11:**
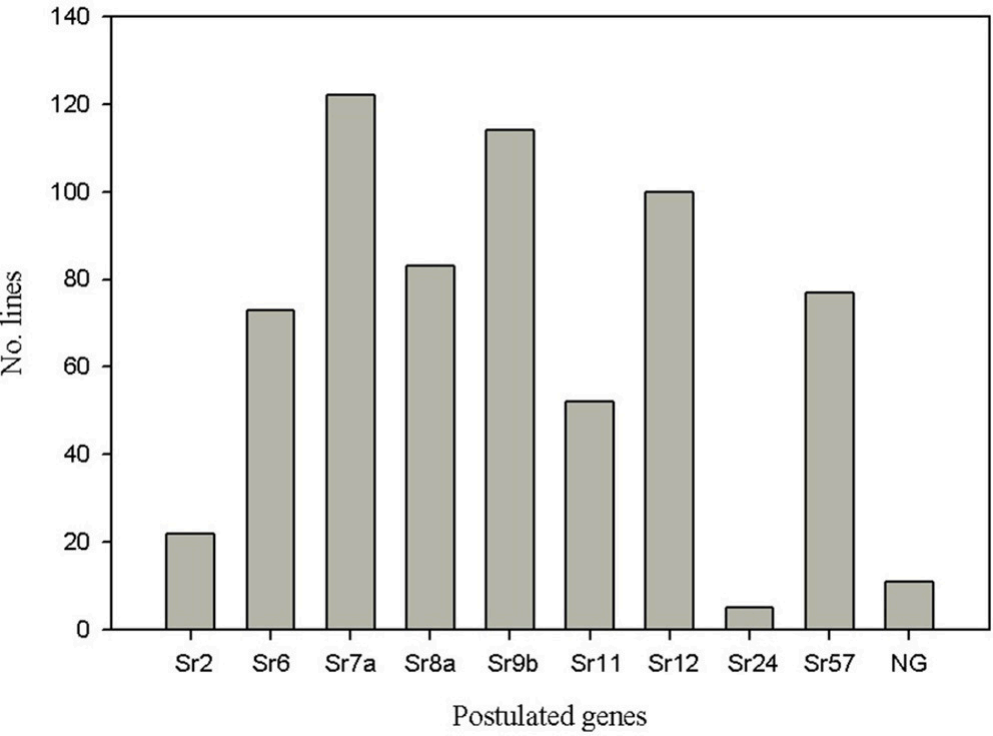
Postulated stem rust resistance genes in the TCAP spring wheat association mapping panel.

We postulated genes/QTL that each line possessed by manually assessing the relationship of its disease response and marker haplotype for each line. We used both seedling IT and field disease responses for *Pgt* races QFCSC, RCRSC, TPMKC, and QTHJC to predict the presence of resistance genes for each line. The postulations were made for 11 previously known genes. A total of 12 lines (5%) lacked any of these genes, and 27 (11%) lines possessed only a single postulated gene (Supplemental Table 8). The remaining 202 (84%) of the lines carried two or more genes (gene combination lines). Over half of the lines (54%) possessed three or more genes. Gene combination lines tended to be composed of various combinations of the most frequent *Sr* genes including *Sr6, Sr7a, Sr8a, Sr9b, S11, Sr12*, and *Sr57* (*Lr34*). However, only line 9,263 from the International Maize and Wheat Improvement Center (CIMMYT) carried the maximum observed nine postulated genes (*Sr6, Sr8a, Sr9b, Sr11, Sr12, Sr24, Sr31*, and *Sr57*). The average number of *Sr* genes per line was 2.8 and varied across breeding programs. Lines from UC Davis possessed an average 1.9 *Sr* genes whereas those from Manitoba possessed an average of 4.4 *Sr* genes. Resistance gene *Sr8a* is known to confer resistance to the virulent race TRTTF (Olivera et al., 2012). Resistance genes *Sr7a* and *Sr11* confer resistance to race TKTTF, a non-Ug99 virulent race that caused the 2013–2014 stem rust epidemic in Ethiopia (Olivera et al., 2015). We also assessed the distribution of each gene in this panel, and found that genes such as *Sr7a* (present in 122 lines), *Sr9b*, and *Sr12* are common in this population and as close to 50% of the population carried each of these genes (Figure 11). Genes *Sr6, Sr8a*, and *Sr57* were present in more than 30% of the population. A total of 21% of the lines in this study were postulated to have *Sr11*. On the contrary, alleles of *Sr2, Sr24, Sr25*, and *Sr31* were rare in this panel (not more than 10% of the lines). The *Sr28*-linked marker *IWB1208* (Babiker et al., 2017) was not available in the dataset from Triticeae Toolbox.

## Discussion

Though several studies have evaluated seedling and/or field response of wheat association mapping panels to *Pgt* isolates (Yu et al., 2011, 2012; Letta et al., 2014; Zhang et al., 2014; Gao et al., 2017; Mihalyov et al., 2017; Muleta et al., 2017), only one has evaluated field response of the wheat population studied to more than one *Pgt* race (Chao et al., 2017). In that study, field response of a bulk of six *Pgt* isolates in Minnesota was compared to response to a bulk of *Pgt* isolates in Ethiopia, and differences between these datasets could be due to environment and/or race. This study is the first to use GWAS to evaluate race-specificity, separate from the effect of environment, of stem rust field response. Our finding often negative and sometimes weak correlations between response to different *Pgt* races highlights the abundance of race-specific resistance and the inability to apply results derived from one *Pgt* race to others. Our finding of usually moderate correlations of seedling and field stem rust responses within each race combined with our finding that usually only the most significant QTL detected between seedling and field tests were consistent also has an important implication: weakly-associated QTL detected in seedling assays by GWAS likely do not translate to effective QTL in the field. As resistance in the field is most desired, caution should be employed when interpreting results from GWAS using seedling assays.

The negative correlations between field resistance to various *Pgt* races, especially between races QFCSC and QTHJC, is a striking finding of our study. This suggests that selecting for increased resistance to one race will actually result in selecting for increased susceptibility to another race. In order to test if QTL were significantly associated with each other, we used Chi-square independence tests. The results indicated that QTL associated with QFCSC and QTHJC resistance were not independent of each other (data not shown). The lack of correlation between Ug99 field data and response to any of the races used in this study suggests that stem rust evaluations to North American *Pgt* isolates cannot be used as a proxy for predicting stem rust response in Africa and may not be applicable for predicting response to future emerging virulent *Pgt* strains.

### Phenotypic variability for resistance to stem rust

The purpose of evaluating the TCAP AM panel for response to *Pgt* races QTHJC, RCRSC, QFCSC, and TPMKC was to facilitate GWAS in order to characterize the genetics of resistance. Also, MTAs identified through this effort can be used for combining multiple genes/QTLs in breeding programs. Analysis of variance showed significant differences among the genotypes in phenotypic resistance against all four *Pgt* races both in seedling evaluations and in the field.

### Population structure and genetic relatedness of the TCAP AM panel

For the current AM panel, all analysis methods considered here (model-based, principal component, and cluster analyses) revealed that there were two major groups that broadly agree with geographic origins of the genotypes. A similar result was also reported previously for the same panel (Bajgain et al., 2015).

### Seedling marker-trait associations (MTA)

With the single-locus mixed model, initially the candidate QTL were identified using a relaxed threshold level with *p*-value of 0.001 without adjusting for multiple comparisons. Although a large number of MTAs (11–28) were detected for each race, there were a total of 25 QTL regions after grouping of MTAs within 5 cM as one QTL region. Out of these, QTL regions detected on chromosome arm 2DS (36–40 cM with four markers) for race QTHJC, on chromosome arm 4AL (144.38–145.19 cM with markers *IAA3545* and *IWA1505*) for race RCRSC were significant at FDR value of 10%. However, with the MLMM, marker *D_contig73920_552* (*IWB17135*) (2DS, 40.05 cM) was strongly associated with QTHJC resistance. Similarly, *IWA1505* (4AL at position 145.19 cM) was the most significantly associated marker with resistance against race RCRSC.

Although none of the candidate QTL regions passed multiple comparisons tests for race QFCSC and TPMKC, with the single-locus model, marker *Excalibur_c1066_303* (1AL at position 102.32 cM) showed strong association with resistance against QFCSC with the MLMM. Similarly, markers *Bobwhite_c18540_97* (*IWB1190*) on chromosome arm 2BL (119.07 cM) and *IWA2415* (2DS, 40.05 cM) were strongly associated with TPMKC resistance with joint-explained phenotypic variation of 16.94%.

The integrated map developed by Maccaferri et al. (2015) was used a reference for comparing our results with previously reported QTL. On chromosome 1A, QTL for QFCSC (at position 102.32 cM) that was detected with marker *Excalibur_c1066_303* (integrated map position 110.01 cM) may be similar to a previously reported stem rust resistance QTL in the region. DArt maker *wpt-0128* (125 cM integrated map position) that flanked stem rust resistance in the PBW343/Kingbird population (Bhavani et al., 2011) is about 15 cM away from the map location of marker *Excalibur_c1066_303* that was associated with QFCSC resistance in the current study. However, other stem rust resistance-associated DArt markers on 1AL such as *wpt-734078* in Bhavani et al. (2011) and *wpt-6869* in Rouse et al. (2014) were mapped on proximal end of chromosome 1AS on the integrated map (Maccaferri et al., 2015). Another study indicated that diagnostic markers for *Sr1RS*^*Amigo*^ on a rye-wheat translocation (T1AL.1RS) were associated with QFCSC, RCRSC, and TPMKC resistance at the seedling stage (Zhang et al., 2014). We also detected a moderate QTL conferring resistance against QFCSC on chromosome arm 1BL (integrated map position 114.13 cM) with marker *IWB40197*. The SNP marker *IWA435* (1B, integrated map position 60.13 cM) identified as a reliable marker to discriminate between *Sr31* (1BL.1RS) and *Sr1RS*^*Amigo*^ (1AL.1RS) by Mihalyov et al. (2017). We used the data from this marker to postulate the presence of *Sr31* (Supplemental Table 8). However, there appears to be one false-positive as line Selkirk clearly does not possess *Sr31* based on susceptible seedling responses to races TRTTF and TKTTF (Bajgain et al., 2015). Association of marker *IWA435* with the four races was also tested and we found that it did not pass the significance threshold level (*P* < 0.001). The association was only significant at *p-value* < 0.05 for RCRSC at the seedling stage. We do not expect either *Sr31* or *Sr1RS*^*Amigo*^ on wheat-rye translocations to be more frequent than 5% in this panel. Therefore, we expect the QTL on 1A effective to QFCSC to be similar to the QTL reported in the Kingbird population or possibly novel.

Marker *BobWhite_c18540_97* (*IWB1190*) on chromosome 2B (119.07 cM and integrated map position 242.14 cM) that was significantly associated with TPMKC resistance mapped at a similar position as the SSR marker *wmc332* (integrated position 242.14 cM) that was linked with major gene *Sr28* on 2BL (Rouse et al., 2012). However, *Sr28* does not confer resistance to TPMKC (Rouse et al., 2012). The haplotype of *IWB1190* in combination with *Sr6* gene-linked marker *IWA2415* also explained only 16.94% of phenotypic variation for race TPMKC seedling response. Moreover, the SNP marker *IWA840* (integrated map position 222.71 cM) recently reported to be linked with *Sr28* (by Mihalyov et al., 2017) is about 20 cM proximal to the QTL-linked marker *IWB1190*, and its associations with all four races are not significant in the current work.

Although the MLMM resolved the association signals detected within a 6 cM segment on chromosome arm 2DS for QTHJC and TPMKC, the single model analysis enabled the comparison of detected QTL across different studies. The MLMM-identified markers, *IWB17135* for QTHJC and *IWA2415* for TPMKC resistance both at the same position (2DS, 40.05 cM) were mapped 16 and 6 cM away, respectively, from the SSR marker *wmc453* (78.5 cM) which was 1.1 cM distal to *Sr6* (Tsilo et al., 2009). With the single model, among the markers within a 6 cM segment on 2DS, markers *Kukri_c20972_618* (*IWB42406*, integrated position 78.7 cM) and Excalibur*_c39215_100* (*IWB26013*, integrated position 74.7 cM) were strongly associated (FDR *P* < 0.1) with QTHJC resistance. These markers were 0.2 cM distal to *wmc453* and 3.8 cM proximal to *wmc453*, respectively. Given that the LD among all markers on chromosome 2DS within the range 34–40 cM is strong (average *r*^2^ = 0.74), *Sr6* may be flanked by these two SNP markers. Gene *Sr6* confers resistance against the predominant *Pgt* races in Unites States such as TPMKC, QFCSC, MCCFC, and QCCJB (McVey et al., 2002; Jin, 2005). Zhang et al. (2014) also found that *Sr6* confers resistance against race QFCSC and TPMKC, but not for race QTHJC contrary to our seedling QTHJC result. Variable levels of virulence or avirulence to *Sr6* have been observed in *Pgt* isolates (Watson and Luig, 1968), so it is possible that *Sr6*, though not qualitatively effective to race QTHJC, does confer a significant quantitative resistance effect to race QTHJC under specific environmental conditions (Forsyth, 1956).

Marker *IWA1505* (4AL at position 145.19 cM) consistently showed strong association with race RCRSC resistance. Documented resistance genes on 4AL are *Sr7* (McIntosh et al., 1995) and *SrND643*, which may be an allele of *Sr7* (Basnet et al., 2015). Comparing the map position of *IWA1505* (integrated map position 180.86 cM) with the positions of SSR markers (*Xgwm350* and *Xwmc219*) that flanked *SrND643* indicated that *IWA1505* was 2.26 cM proximal to *Xwmc219* and 0.64 cM distal to the 2nd closest SSR marker proximal to *SrND643*. This marker was also mapped 12.47 distal and 8.94 cM proximal to SSR marker *barc78* (168.39 cM) and *wmc313* (199.8 cM) that flanked the *Sr7* locus (Turner et al., 2016), respectively. Marker *barc78* was also associated race JRCQC resistance in a durum wheat association mapping panel (Letta et al., 2014). We expect that the RCRSC resistance locus corresponds to *Sr7a* in our study based on the overlap of markers postulated to be associated with *Sr7a* in Bajgain et al. (2015) and proximity to markers linked to *Sr7a* in Turner et al. (2016).

### Field resistance QTL

Markers such as *GENE-0966_173* and *Excalibur_c76665_98* (*IWB28807*) on chromosome arm 2BL (consensus map position 109.25 cM; integrated position 201.209 cM) and unmapped marker *IAAV2323* consistently associated with response to race QFCSC. Marker *IAAV2323* is in linkage equilibrium (*r*^2^ < 0.1) with all other markers involved in MTAs and it is most likely a different locus. Those two markers on chromosome 2B are very close (1.84 cM) to the DArt marker *wPt-8460* (integrated position 199.37 cM), a marker linked to *Sr9h* gene both in biparental mapping (Rouse et al., 2014) and association mapping (Yu et al., 2014). Other major genes such as *Sr47* and *Sr28* are also nearby on chromosome arm 2BL (Singh et al., 2011; Yu et al., 2014), but are unlikely candidates as they are either derived from alien species and not present in this panel or they are not effective to QFCSC (*Sr28*). We expect the QTL on 2BL that confers resistance to QFCSC to likely be an allele at the *Sr9* locus based on map position. Of the described *Sr9* alleles, *Sr9b* is likely the allele conferring resistance to race QFCSC that is associated with markers *GENE-0966_173* and *Excalibur_c76665_98*. The similar detection of this locus on 2BL in response to TPMKC fits the *Sr9b* hypothesis based on race-specificity of this QTL compared to *Sr9b*.

On chromosome 4A, the marker *IAAV3545* (4A at 144.38 cM) explained 16.23–21.53% of the phenotypic variation and consistently showed the strongest association with resistance against RCRSC across models, traits, and growth stages (seedling and adult plant). Since the *Sr7b* allele is not effective to RCRSC, the resistance at this locus most likely conferred by the *Sr7a* allele. A previous association study included all the four races (QFCSC, QTHJC, RCRSC, and TPMKC) also showed that *Sr7a* was associated with race RCRSC resistance but not with the resistance against the remaining three races (Zhang et al., 2014).

The QTL region that was detected on 2DS (34–40 cM) with several markers including *D_contig73920_552* (*IWB17135*) showed strong associations with QTHJC and TPMKC resistance both at seedling and adult plant stages. Comparing the map positions markers from different platforms and LD analysis, the QTL detected on chromosome arm 2DS is in the region of the previously known major gene *Sr6*. This indicates the possibility of tracking *Sr6* in breeding programs by converting those chip-based SNPs that showed strong associations (e.g., *D_contig73920_552*) into PCR-based SNP markers. Unmapped markers *IWA760, RAC875_c7319_195* and Excalibur*_c3574_607* showed strong associations with race QTHJC resistance across traits and models for field resistance in this study. The 1st two markers in perfect LD (*r*^2^ = 1) and had moderate LD (*r*^2^ = 0.3) with a marker mapped at 22.46 cM on chromosome 2DS. However, both are in linkage equilibrium (*r*^2^ = 0.01) with the 3rd marker. Interestingly marker *Excalibu*r*_c3574_607* showed strong LD that ranged from 0.5 to 0.94 (average *r*^2^ = 0.76) with markers within the range 34–40 cM on chromosome 2DS indicating this marker is linked with *Sr6*.

Although numerous moderate MTAs were detected for each race in the current study, the consistently-detected QTL across traits, plant growth, or QTL detection methods with strong association signals were mainly in the regions of known major stem rust resistance genes *Sr6, Sr7a*, and *Sr9b*. In addition, the majority of the detected MTAs are race-specific indicating the absence of broadly effective genes in this population (Bajgain et al., 2015). However, based on our gene postulation, there are lines that carry combinations of genes that confer resistance to both North American and virulent African and West Asian *Pgt* races (e.g., TRTTF and TKTTF). Our data do not allow postulation of all *Sr* genes present in this panel. For example, we postulated the presence of zero *Sr* genes in wheat line Marquis. Previous studies demonstrated that Marquis possesses at least four *Sr* genes (*Sr7b, Sr18, Sr19*, and *Sr20*; Knott, 1965). These and other *Sr* genes were not detected in our study because of any of the following reasons: ineffectiveness to the *Pgt* races assayed, low frequency in the panel, and lack of data from additional *Pgt* isolates that would allow accurate postulation. The *Sr* genes that we postulated are effective to one or more of the modern North American *Pgt* races. Our gene postulation data could be used to guide breeders seeking donor lines for particular *Sr* genes. For example, *Sr* genes *Sr7a, Sr9b*, and *Sr12* were frequently present in the Minnesota and Manitoba lines, but other potentially valuable *Sr* genes including *Sr2* and *Sr25* were absent. Overall our data suggest the presence of *Sr* gene combinations in the majority of North American spring wheat breeding lines. Though many of these *Sr* genes are race-specific, combination of them results in resistance to multiple North American *Pgt* isolates.

## Author contributions

EE: conducted data analyses and prepared the first draft; MP: provided seeds of the mapping panel and reviewed the manuscript; MR: conceived the study, designed, and conducted both field and greenhouse experiments, and reviewed the manuscript.

### Conflict of interest statement

The authors declare that the research was conducted in the absence of any commercial or financial relationships that could be construed as a potential conflict of interest.

## Supplementary material

Supplemental Figure 1 Seedling infection type phenotypic mean (BLUPs) distribution for four the four stem rust races (QTHJC, TPMKC, QFCSC, and RCRSC).

Supplemental Figure 2 Phenotypic correlation coefficients among four races based on the infection type.

Supplemental Figure 3 Phenotypic distribution of field infection response mean (BLUPs).

Supplemental Figure 4 Phenotypic distribution of field disease severity mean (BLUPS).

Supplemental Figure 5 Phenotypic correlation coefficients both seedling data and field data sets.

Supplemental Figure 6 Phenotypic correlation between disease response of North American stem rust races evaluated in Rosemont, MN, and Ug99 evaluated in Kenya and Ethiopia.

Supplemental Figure 7 Graph showing optimum number of population structure of TCAP Spring wheat association mapping panel.

Supplemental Figure 8 Population structure plot for the TCAP Spring wheat Association mapping panel.

Supplemental Figure 9 Q-Q plot for four races based on seedling infection type genome-wide association analysis.

Supplementary Table 1 Isolates of *Puccinia graminis* f. sp. *tritici* used in field and seedling assays and their reaction to differential wheat stem rust resistance genes.

Supplementary Table 2 Variance components for four stem rust races for infection type at seedling stage for 250 entries evaluated in Greenhouse.

Supplementary Table 3 Cluster groups of spring wheat association mapping panel based on model-based population structure analysis.

Supplementary Table 4 Marker-trait associations identified for four stem rust races at seedling stage.

Supplementary Table 5 Marker-trait associations for race QFCSC-IR.

Supplementary Table 6 Marker-trait associations for race RCRSC-SEV.

Supplementary Table 7 Marker-trait associations for race TPMC-IR.

Supplementary Table 8 Gene postulation for spring wheat association mapping panel based on phenotypic and marker data.
