# Supplementary figures and images for: A Genome-Wide Association Study of Field and Seedling Response to Individual Stem Rust Pathogen Races Reveals Combinations of Race-Specific Genes in North American Spring Wheat
Source: Front Plant Sci. 2018 Jan 30;9:52. doi: 10.3389/fpls.2018.00052 (PMC5797647; doi:10.3389/fpls.2018.00052)

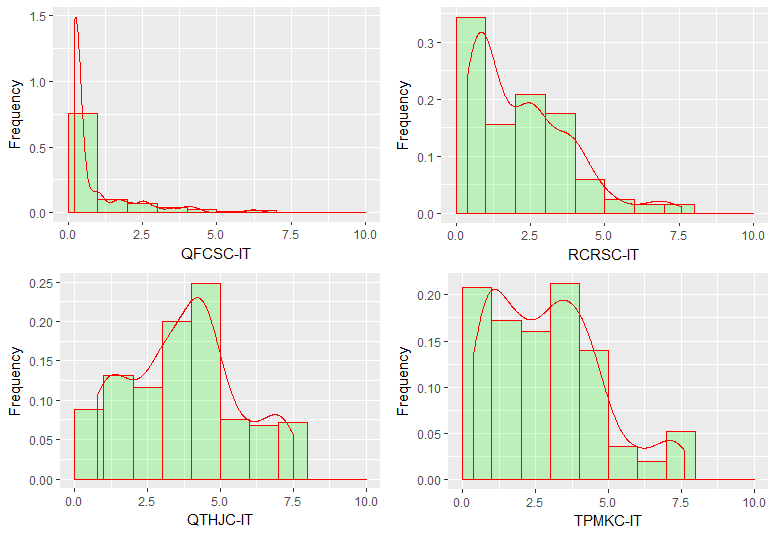

Supplement: Supplemental Figure 1 — Seedling infection type phenotypic mean (BLUPs) distribution for four the four stem rust races (QTHJC, TPMKC, QFCSC, and RCRSC). [file Image1.TIFF]

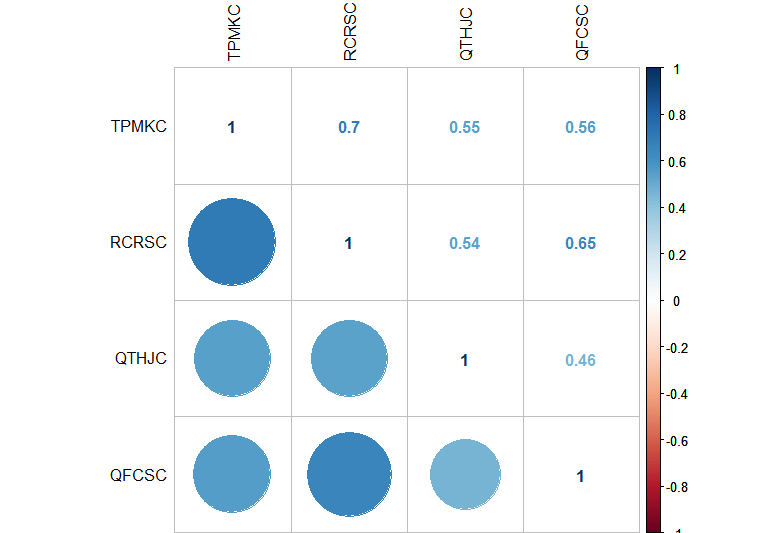

Supplement: Supplemental Figure 2 — Phenotypic correlation coefficients among four races based on the infection type. [file Image2.TIFF]

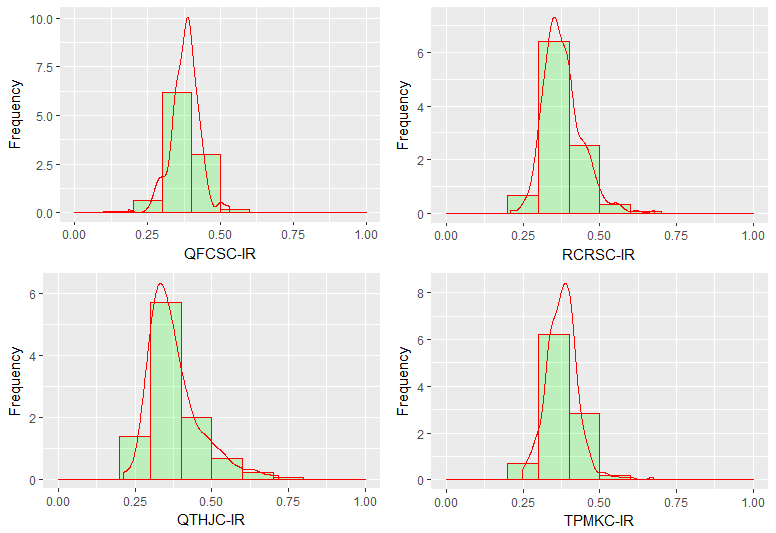

Supplement: Supplemental Figure 3 — Phenotypic distribution of field infection response mean (BLUPs). [file Image3.TIFF]

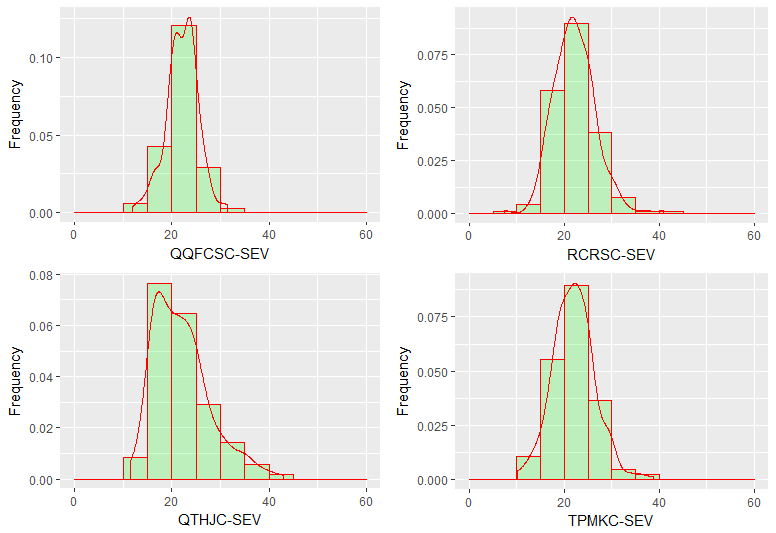

Supplement: Supplemental Figure 4 — Phenotypic distribution of field disease severity mean (BLUPS). [file Image4.TIFF]

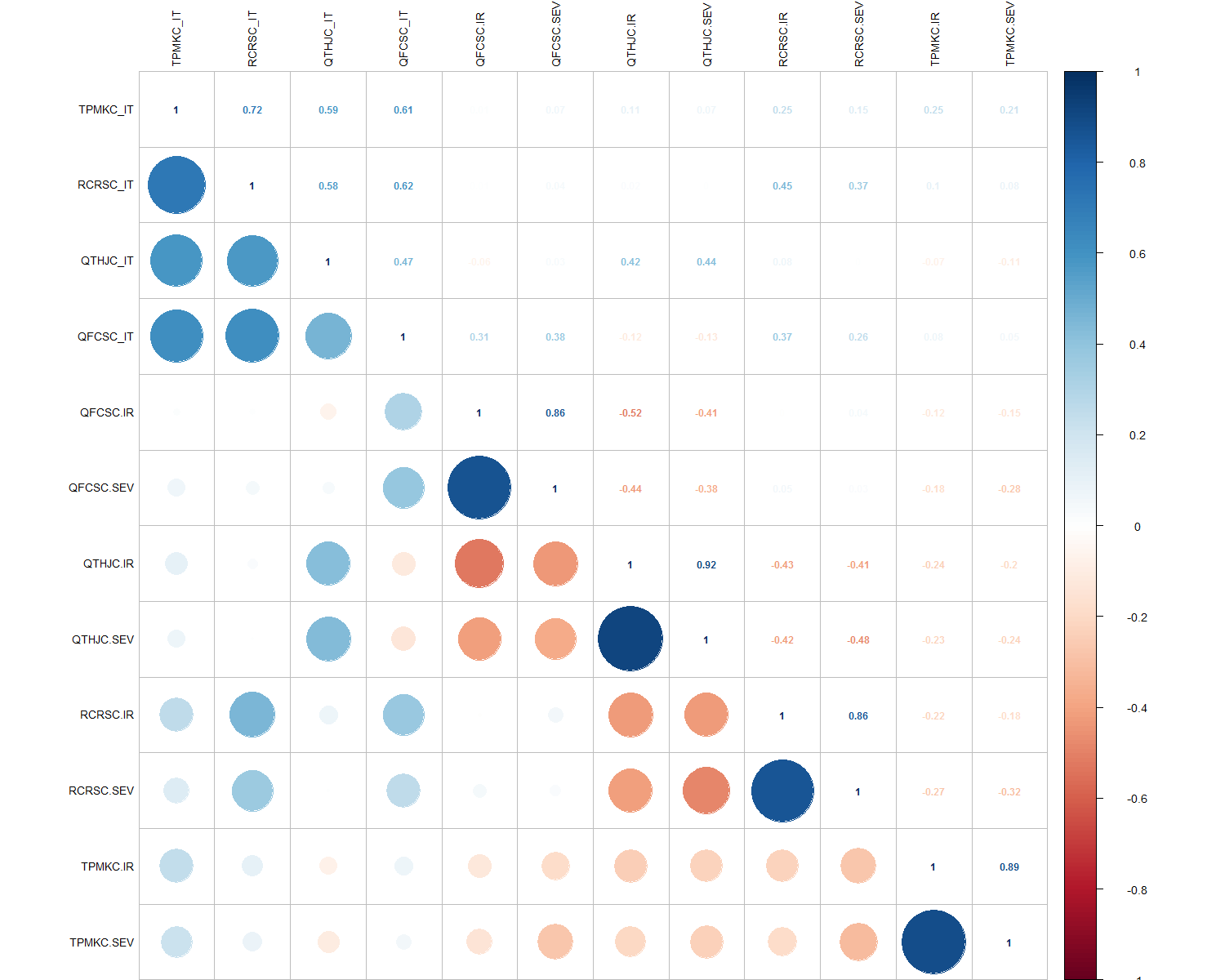

Supplement: Supplemental Figure 5 — Phenotypic correlation coefficients both seedling data and field data sets. [file Image5.PNG]

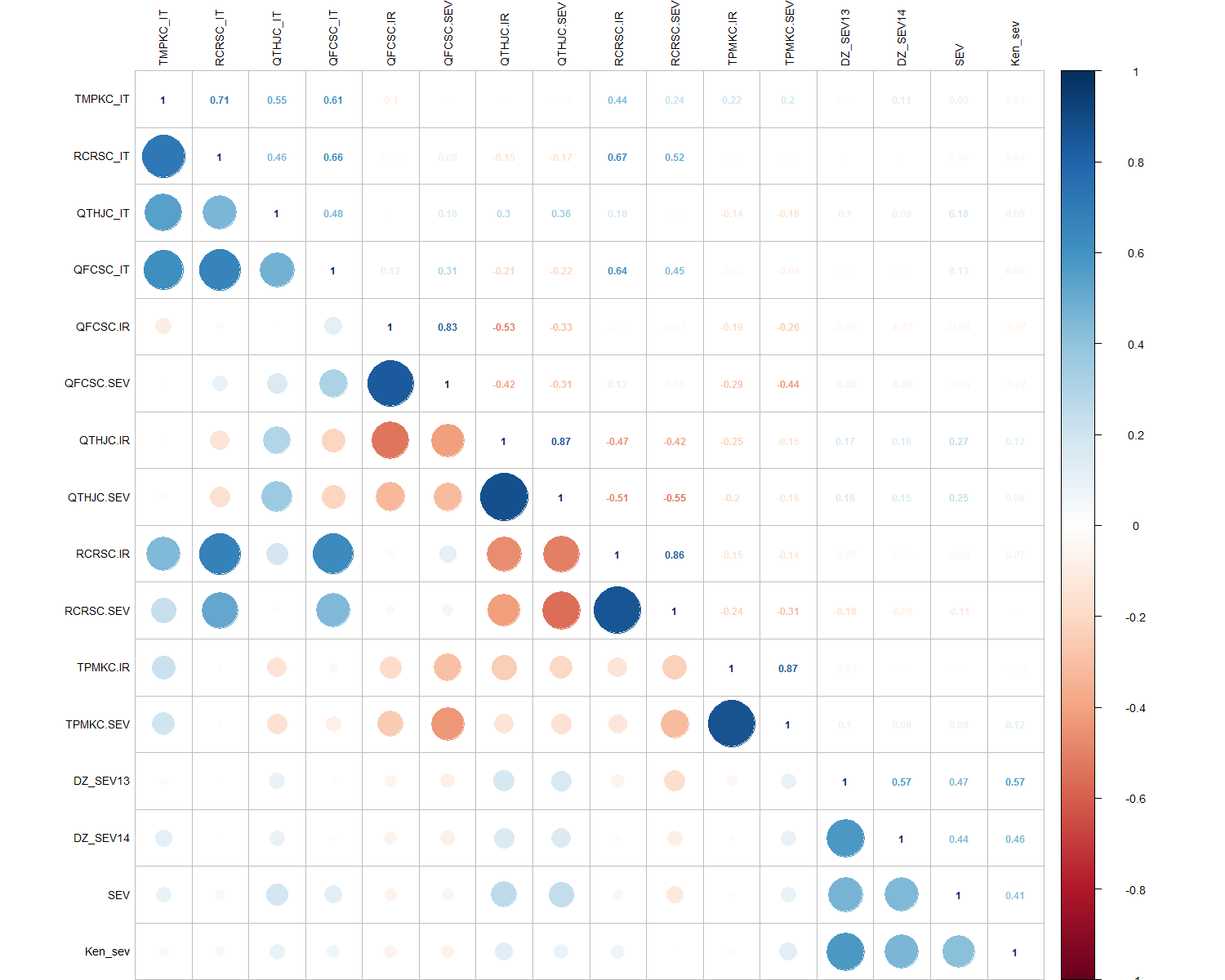

Supplement: Supplemental Figure 6 — Phenotypic correlation between disease response of North American stem rust races evaluated in Rosemont, MN, and Ug99 evaluated in Kenya and Ethiopia. [file Image6.PNG]

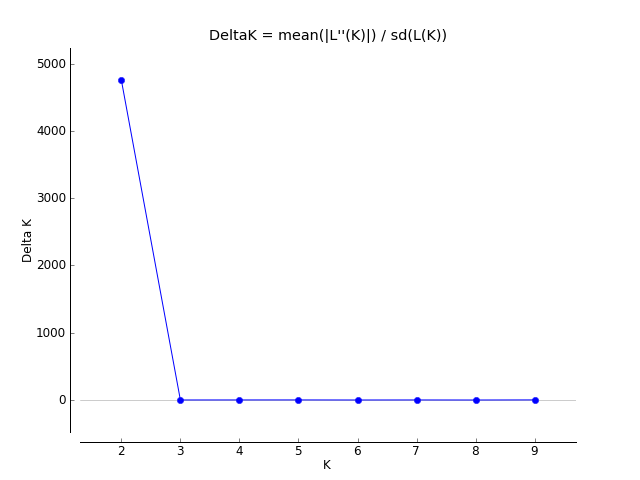

Supplement: Supplemental Figure 7 — Graph showing optimum number of population structure of TCAP Spring wheat association mapping panel. [file Image7.PNG]

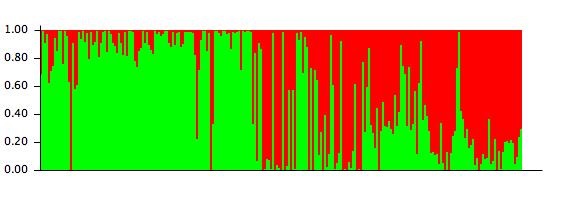

Supplement: Supplemental Figure 8 — Population structure plot for the TCAP Spring wheat Association mapping panel. [file Image8.JPEG]

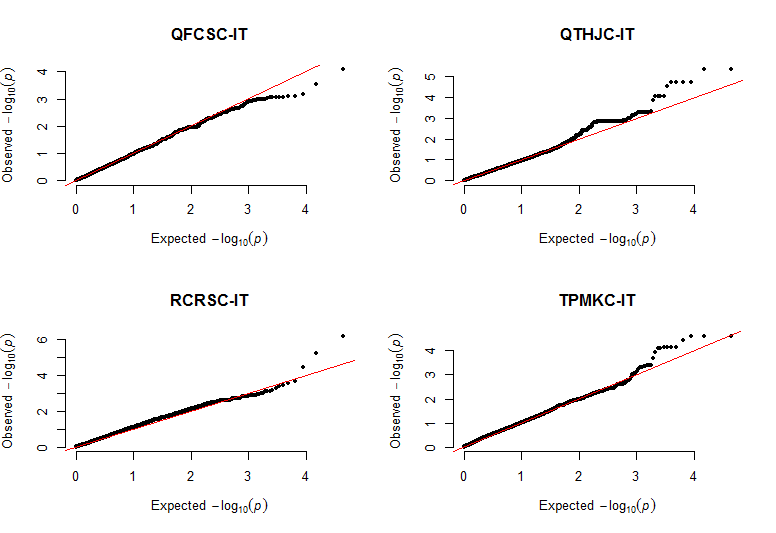

Supplement: Supplemental Figure 9 — Q-Q plot for four races based on seedling infection type genome-wide association analysis. [file Image9.TIFF]
