# Supplementary material for: A Genome-Wide Association Study of Field and Seedling Response to Individual Stem Rust Pathogen Races Reveals Combinations of Race-Specific Genes in North American Spring Wheat
Source: Front Plant Sci. 2018 Jan 30;9:52. doi: 10.3389/fpls.2018.00052 (PMC5797647; doi:10.3389/fpls.2018.00052)
Supplement: Supplementary Table 2 — Variance components for four stem rust races for infection type at seedling stage for 250 entries evaluated in Greenhouse. [file Table2.docx]

| Race | Entry | Block | Residual |
| --- | --- | --- | --- |
| TPMKC | 3.82*** | 0.00ns | 1.15*** |
| RCRSC | 3.09*** | 0.05ns | 1.27** |
| QTHJC | 4.23*** | 0.32ns | 2.19*** |
| QFCSC | 2.05*** | 0.08ns | 1.37*** |

Supplemental Table 2. Variance components for four stem rust races for infection type at seedling stage for 250 entries evaluated in Greenhouse.
